# Distribution of subcutaneous and intermuscular fatty tissue of the mid-thigh measured by MRI—A putative indicator of serum adiponectin level and individual factors of cardio-metabolic risk

**DOI:** 10.1371/journal.pone.0259952

**Published:** 2021-11-15

**Authors:** Eva Maria Hassler, Hannes Deutschmann, Gunter Almer, Wilfried Renner, Harald Mangge, Markus Herrmann, Stefan Leber, Manuela Michenthaler, Alexander Staszewski, Felix Gunzer, Richard Partl, Gernot Reishofer

**Affiliations:** 1 Division of Neuroradiology, Vascular and Interventional Radiology, Department of Radiology, Medical University Graz, Graz, Austria; 2 Clinical Institute of Medical and Chemical Laboratory Diagnostics, Medical University Graz, Graz, Austria; 3 BioTechMed Graz, Graz, Austria; 4 Department of Radiology, Medical University Graz, Graz, Austria; 5 Department of Therapeutic Radiology and Oncology, Medical University Graz, Graz, Austria; Università degli Studi di Milano, ITALY

## Abstract

Obesity and metabolic syndrome (MetS) are associated with hypoadiponectinemia. On the contrary, studies revealed correlations between the amount of subcutaneous adipose tissue (SAT) and higher serum adiponectin levels. Furthermore, independent association of intermuscular adipose tissue (IMAT) deposit in the thigh with cardiometabolic risk factors (including total blood cholesterol, low-density lipoprotein (LDL), and triglycerides), and decreased insulin sensitivity, as MetS components, are sufficiently described. The combined relationship of thigh IMAT and SAT with serum adiponectin, leptin levels, and cardiometabolic risk factors have not been investigated till date. Since both SAT and IMAT play a role in fat metabolism, we hypothesized that the distribution pattern of SAT and IMAT in the mid-thigh might be related to adiponectin, leptin levels, and serum lipid parameters. We performed adipose tissue quantification using magnetic resonance imaging (MRI) of the mid-thigh in 156 healthy volunteers (78 male/78 female). Laboratory measurements of lipid panel, serum adiponectin, and leptin levels were conducted. Total serum adiponectin level showed a significant correlation with the percentage of SAT of the total thigh adipose tissue (SAT/ (IMAT+SAT)) for the whole study population and in sex-specific analysis. Additionally, SAT/(IMAT+SAT) was negatively correlated with known cardiometabolic risk factors such as elevated total blood cholesterol, LDL, and triglycerides; but positively correlated with serum high-density lipoprotein. In multiple linear regression analysis, (SAT/(IMAT+SAT)) was the most strongly associated variable with adiponectin. Interestingly, leptin levels did not show a significant correlation with this ratio. Adipose tissue distribution in the mid-thigh is not only associated to serum adiponectin levels, independent of sex. This proposed quantitative parameter for adipose tissue distribution could be an indicator for individual factors of a person`s cardiometabolic risk and serve as additional non-invasive imaging marker to ensure the success of lifestyle interventions.

## Introduction

Metabolic syndrome (MetS) is a clustering of several medical conditions such as dyslipidemia, insulin resistance, hypertension, and adiposity [1–3]. Some studies observed lower concentrations of serum adiponectin with MetS, and an inverse association with most MetS components and serum adiponectin levels in adolescents [4–8]. A higher serum adiponectin level showed a beneficial effect on glucose and lipid metabolism and improves insulin sensitivity via endoplasmic reticulum stress induced by autophagy, in skeletal muscle cells [6]. Moreover, adiponectin is considered to have an atheroprotective effect in adults and even in adolecents [8–12] and a recent study indicated the prognostic value of serum adiponectin level for coronary artery disease [13].

Adiponectin, one of hundreds of adipokines, was identified in the last decades [14,15]. The first adipokine to be discovered, leptin, regulates hedonistic drives (hunger and satiety) in the central nervous system and thus, energy homeostasis. In contrast to adiponectin, increased leptin levels usually occur in obesity, and here leptin resistance is also assumed [16,17]. The high levels occurring in leptin resistance are also associated with an increase in proinflammatory cytokines. They increase the risk of developing diabetes and infertility and are linked to an elevated risk of developing atherosclerosis [17–20]. Leptin and adiponectin increase fatty acid (FA) oxidation in muscle cells and decrease triglyceride storage in the muscle [21–24]; one possible explanation for the insulin-sensitizing effects of these cytokines [21].

The subcutaneous adipose tissue (SAT) compartment functions as a metabolic buffer for excess lipid storage [25,26]. The amount of SAT mostly measured in the abdominal region is associated with higher serum adiponectin levels due to the higher adiponectin expression in the SAT compartment [25,27]. Pilz et al. found a relationship between SAT in the thigh region and serum adiponectin levels in juveniles [12]. This leads to the assumption that SAT in the thigh region can have a positive effect on glucose metabolism and an indirect atheroprotective effect via higher serum adiponectin levels. However, Kwon et al. [28] also showed a protective effect of the abdominal SAT amount against some MetS components, such as high blood pressure, high fasting glucose, and high serum triglycerides (TG). Additionally, recent distinct gene expression patterns have been described for SAT and IMAT, in trying to identify the different functions of AT types [29].

Some studies indicate that IMAT, which is the visible AT between the muscle fibers and beneath the fascia, is independently correlated with cardiovascular risk factors [30–33]. In particular, its influence on glucose metabolism seems to be undisputed [31,34,35]. In addition to the amount of IMAT, the location seems to play an important role. IMAT in the thigh muscles, in particular, appears to be a stronger predictor of cardiovascular risk than IMAT in calf muscle tissue [30]. Furthermore, AT insulin resistance in muscle tissue plays a key role in the development of metabolic and histological abnormalities in obese patients with non-alcoholic fatty liver disease (NAFLD) [36].

Although the exact mechanisms by which IMAT promotes insulin resistance are unknown, the initial report by Goodpaster et al. [32] suggests that IMAT may lead to insulin resistance from reduced blood circulation in muscle tissue or decreased insulin diffusion capacity. This mechanism can be explained by the increased local free FA concentration [37]. Additionally, the expression of ADIPOR 1 and ADIPOR2 mRNA in thigh muscles of Tibetian chickens correlated positively with IMAT in males [38].

Measuring the total amount of adipose tissue (SAT and IMAT) is only possible using radiological methods that can quantify subcutaneous fat deposits, intramuscular infiltration, visceral adipose tissue and AT deposits around vascular structures. Since CT exposes the patients to ionizing radiation [31], it can only be used if it is clinically indicated. MRI enables proper differentiation between AT and surrounding structures and is therefore, most suitable for the quantification of different adipose tissue compartments [31,39,40].

The Dixon technique is one of the most common MRI techniques for the quantification of AT in different compartments. It allows the generation of water-only (W) and fat-only (F) signals, due to the slightly different resonance frequencies of protons in fat and water, known as chemical shift.

In this study, we used a two-point DIXON technique to quantify the amount of IMAT and SAT in the thigh of 156 volunteers [41]. The aim of our study was to investigate the relationship between the distribution of IMAT and SAT in the thigh region with adiponectin and leptin concentrations, as well as serum lipid parameters, which has not been examined till date. In particular, the influence of IMAT has not yet been completely clarified.

## Materials and methods

### Subjects

This study was approved by the local Ethics Committee of the Medical University Graz; (approval number: EK-Nr. 29–585 ex 16/17). Written informed consent was obtained from all the participants before enrollment. In this cross-sectional study, we recruited 156 volunteers. Exclusion criteria were: known lipid disorders; some metabolic disorders such as diabetes, muscle diseases, neurological disorders; chronic diseases; and a regular intake of cholesterol-reducing medication or hormones. Due to the MRI examination, we excluded all patients with metallic implants and claustrophobia. Prior to MRI, we measured the height, waist circumference, and hip circumference of all volunteers using an inelastic measuring tape. All volunteers were weighed on the same personal scale. The waist/hip and waist/height ratios were calculated using Microsoft Office Excel 2016 (Microsoft Corporation, USA).

All enrolled study participants completed a questionnaire on pre-existing conditions and lifestyles including cardiovascular risk factors like smoking, hypertension according to the guidelines of the European society of cardiology [42]. They indicated the frequency, endurance, and nature of their sporting activities as well as their dietary style. Weekly strength and endurance training times were recorded.

### Magnetic resonance imaging (MRI)

**Fig 1:**
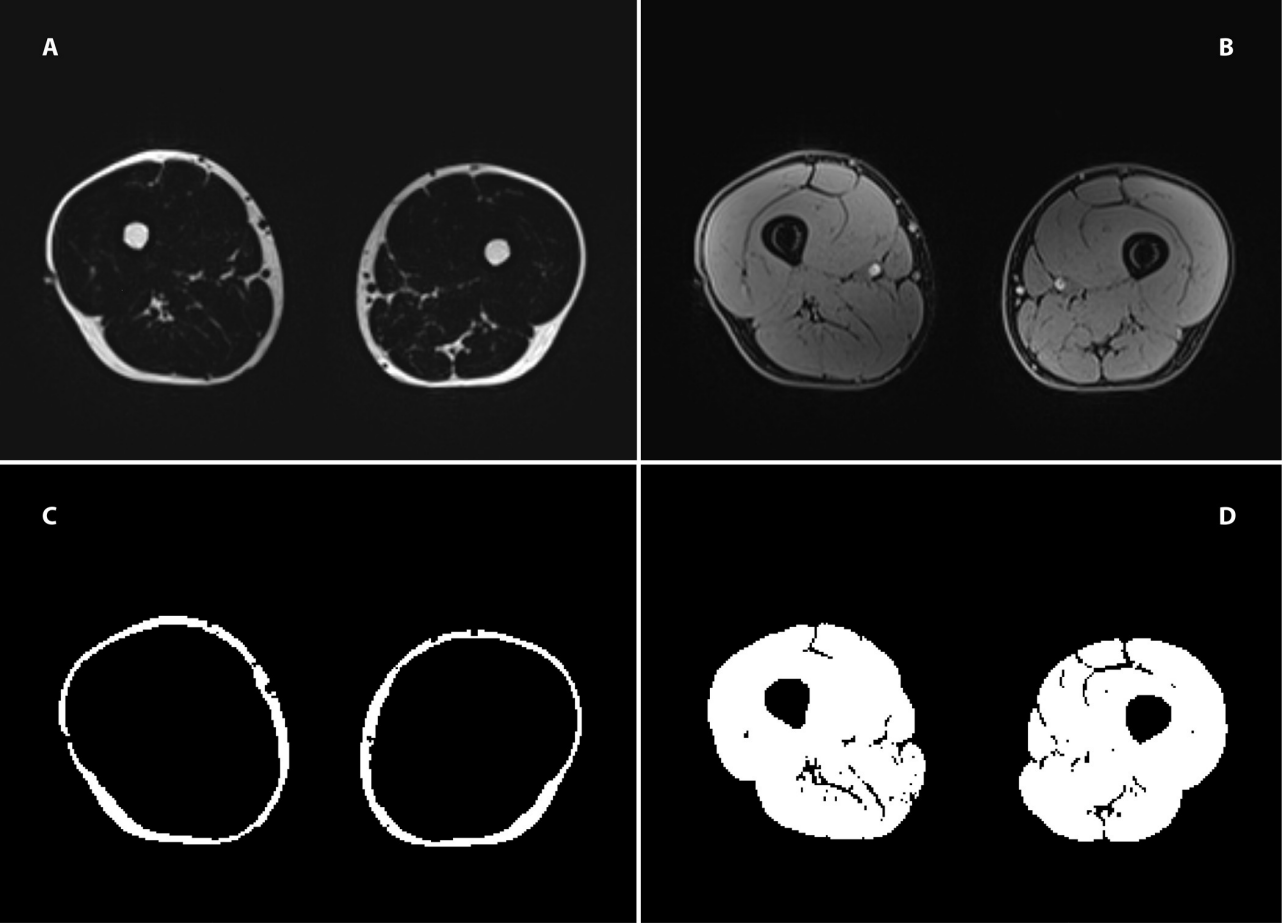
MR images of the thigh. First row: MRI images acquired using the 2-point DIXON sequence. (A) the "fat-only” image; (B) the "water-only” image. Second row: (C) the segmentation result of a patient’s SAT; (D) the segmentation results of the muscle area.

To ensure that a comparable region was measured for every subject using MRI, a marker was positioned on the front thigh using a standardized method developed for ultrasound measurements of SAT [43]. After positioning the marker, MRI of the thigh was performed in the supine position with a 3 Tesla magnetic resonance scanner (Siemens MAGNETOM® Prisma, Siemens Healthcare, Erlangen, Germany) using an eight-channel matrix surface coil. The MRI scan consists of a standard 2- Point DIXON sequence (TR 4.66ms, TE1 1.24ms, TE2 2.47ms, Flip angle 9°, base resolution 288, ISO Voxel Size: 1.4 x 1.4x1.4 mm, PAT 4), generating a fat and water image as shown in Fig 1A and 1B.

### Image processing

The MRI data were analyzed using FIJI, an open-source image processing package based on ImageJ [44]. The slice with the most visible positioning marker was used for the segmentation of IMAT and SAT (Fig 1C and 1D). IMAT was defined as the adipose tissue between the muscle groups and beneath the fascia. A mask for IMAT and SAT was generated and applied to a Dixon fat image. The amount of IMAT and SAT was measured in centimeter squared. We calculated the percentage of SAT of the total thigh AT as follows: SAT/(SAT+IMAT). The fat fraction (FF) was calculated from signal intensities derived from fat-only images (SI_FAT_) and water-only images (SI_WATER_) according to the formula: FF = SI_FAT_/ (SI_FAT_+SI_WATER_). Fig 1 shows the fat-only image (A), water-only image (B), and segmentation results for the SAT and muscle.

### Laboratory analysis

Venous blood samples were collected from every subject prior to MRI measurements, after a 12 h overnight fast. Serum lipid analyses included total blood cholesterol, HDL, LDL, and serum TG. The total cholesterol/HDL quotient was calculated using MS Office Excel 2016 (Microsoft Corporation, USA). The serum adipokine levels of adiponectin and leptin were analyzed using the appropriate Human ELISA Kits® (both from BioVendor, Czech Republic) according to the manufacturer’s instructions. Standards, controls and samples were measured in duplicate. Standard curves were calculated using a four-parameter algorithm. All blood samples were analyzed in the same laboratory using a standardized protocol of frozen serum (− 80°C).

### Statistical analysis

Statistical analyses were performed using RStudio (R version 4.0.2; Integrated Development for R. RStudio, PBC, Boston, MA). Statistical comparison of the subjects’ continuous parameters was accomplished using the two-sided Student’s t-test with Pearson’s linear correlation coefficients for the whole study population. The subjects were separated into groups according to sex. For non-parametric data, the Mann-Whitney-U-Test was utilized. Distribution was tested for normality using the Shapiro–Wilk test. A p-value < .05 was considered statistically significant. Furthermore, we performed a multiple linear regression analysis with adiponectin as a constant, and included BMI, waist/height, and waist/hip as independent variables. For group comparison, the non-parametric Kruskal-Wallis test was performed.

## Results

In total, 156 volunteers (78 males, 78 females; mean±SD, 43.62 ± 11.23, min-max, 21.4–76.3 years) were included in the study. Table 1 shows all anthropometric measurements including height, weight, waist and hip circumferences, segmented areas and ratios of the fat compartments, and laboratory parameters of the entire study population. Table 2 shows the correlation between lipid profile, segmentation results, and anthropometric measurements. All relevant raw data are included in the supporting information of the manuscript (S1 Table).

**Table 1:** Descriptive statistic of the study population including age and anthropometric measurements. Results of the fat segmentation and the laboratory parameters are presented. p-values mark sex differences (Values are reported as mean ± SD).

*Anthropometric measurements*	Total (n = 156)	Females (n = 78)	Males (n = 78)	p-value
**Age (years)**	43.62 ± 11.23	45.59 ± 10.63	41.65 ± 11.53	**.028** *
**BMI (kg/m** ^**2**^ **)**	23.74 ±3.11	22.88 ± 3.27	24.60 ± 2.70	**.005** **
**Waist circumference (cm)**	81.93 ± 10.64	76.65 ± 9.30	87.22 ± 9.20	**< .001** ***
**Hip circumference (cm)**	99.84 ± 7.43	98.26 ±7.88	101.42 ± 6.63	**.007** **
**W/hip ratio**	0.82 ±0.08	0.78 ± 0.06	0.86 ± 0.07	**< .001** ***
**W/height ratio**	0.47 ± 0.06	0.46 ± 0.06	0.48 ± 0.05	**.009** **
***Fat compartments***	*** ***	*** ***	*** ***	*** ***
**SAT (area in cm** ^**2**^ **)**	106.13 ± 55.63	142.87 ± 50.71	69.40 ± 30.39	< .001***
**IMAT (area in cm** ^**2**^ **)**	14.60 ± 9.63	18.24 ± 11.01	10.95 ± 6.21	< .001***
**SAT/(SAT + IMAT**)	0.88 ± 0.05	0.89 ± 0.05	0.87 ±0.05	**.006** **
**Fat fraction**	0.09 ± 0.03	0.09 ± 0.04	0.08 ±0.02	**.004** **
**Muscle cross-sectional area (in cm** ^**2**^ **)**	274.37 ± 65.68	225.23 ± 28.85	323.52 ± 54.38	**< .001** ***
***Lipid profile***	*** ***	*** ***	*** ***	*** ***
**Total blood cholesterol (mg/dL)**	194.52 ± 32.60	199.53 ± 30.51	189.51 ± 34.02	.055
**HDL (mg/dL)**	68.94 ± 17.73	75.94 ± 18.67	61.94 ± 13.60	**< .001** ***
**LDL (mg/dL)**	103.73 ± 31.80	103.77 ± 30.29	103.10 ± 32.36	.9
**Triglycerides (mg/dL)**	115.46 ±88.68	108.05 ± 99.04	122.86 ± 76.87	.3
**Quot(Chol/HDL)**	3.02 ± 1.07	2.78 ± 1.00	3.25 ± 1.08	**.004** **
**Adiponectin (μg/mL)**	11.59 ± 4.17	12.90 ± 4.71	10.28 ± 3.07	**< .001** ***
**Leptin (ng/mL)**	8.65 ± 8.85	9.52 ± 9.45	7.69 ± 8.09	**.2**

Results of the fat segmentation and the laboratory parameters are presented.

p-values indicate sex differences (values are reported as mean ± standard deviation (SD)).

*body mass index (BMI)*, *cholesterol (Chol)*, *high-density lipoprotein (HDL)*, *intramuscular fatty tissue (IMAT)*, *low-density* lipoprotein *(LDL)*, *muscle mass (MM)*, *subcutaneous fatty tissue (SAT)*, *waist (W);*

*p < .*05*

**p < .*01*

***p < .*001; n = 156*.

**Table 2:** Correlation between fat tissue segmentation results, adiponectin, leptin, lipid profile, and anthropometric measurements. Pearson correlation coefficients (r-values) and associated p-values in parentheses are shown.

Adipose tissue segmentation		Adiponectin	Leptin	HDL	Total blood cholesterol	LDL	Triglycerides	Chol/HDL
SAT/(IMAT+SAT)		**.32** *** **(< .001)**	.04 (.600)	.33*** **(< .001)**	**-.17** ** **(.037)**	-.23** **(.004)**	**-.27** ** **(.001)**	-.36*** **(< .001)**
IMAT/(IMAT+SAT)	**-**	**-.32** *** **(< .001)**	-.04 (.600)	**-.33** *** **(.001)**	.17** **(.037)**	.23** **(.004)**	.27** **(.001) **	.36*** **(< .001)**
SAT		**.3** *** **(< .001)**	.03 (.700)	.06 (0.400)	.19** **(.020**)	.13 (.100)	.07 (.4)	.08 (.3)
IMAT	-	-.05 (.600)	.05 (.600)	**-.20** ** **(.010)**	**.27** *** **(< .001**)	**.24** ** **(.003)**	.24** **(.003)**	**.32** *** **(< .001)**
Cross-sectional area MM	**-**	**-.26** *** **(< .001)**	-.18** **(.030**)	**-.32** *** **(< .001**)	.16 (.050)	.01 (.900)	< .01 (.990)	**.20** ** **(.010)**
Fat fraction		.06 (.400)	.05 (.600)	-.02 (.800)	.22** **(.005**)	.17 * ** **(.040)**	.17** **(.030)**	.14 (.09)
**Lipid profile**	** **	** **	** **	** **	** **	** **	** **	
HDL		**.29** *** **(< .001**)	.07 (.400)	-	-	-	-	
Total blood cholesterol		.09 (.3)	-.06 (.400)	.01 (.090)	-	-	-	
LDL	-	.01 (.9)	-.07 (.400)	.86*** **(< .001)**	.86*** **(< .001)**			
Triglycerides	-	.12 (.15)	-.02 (.800)	**.36** *** **(< .001)**	.36*** **(< .001)**	** **		
Chol/HDL	**-**	**.20** *** **(.01)**	-.06 (.400)	-.06 (.400)-	**-**	.69*** **(< .001)**	** **	
**Anthropometric measurements**			** **	** **	** **	** **	** **	
Waist/Height	**-**	**.16** * (**.048)**	-.01 (.9)	-.31*** **(< .001**)	** **	.34*** **(< .001)**	.53*** **(< .001)**	
Waist/Hip	**-**	.31*** **(< .001**)	-.01 (.97)	-.18** (**.024**)	** **	.28*** **(< .001)**	**.43** *** **(< .001)**	
BMI	**-**	.18 ** (**.03)**	.10(.2)	**-.20** ** **(.009)**	** **	**.25** ** **(.001)**	.49*** **(< .001)**	
** **	** **	** **	** **	** **	** **	** **	** **	
**Adipose tissue segmentation**	** **	**Waist/Height**	** **	**Waist/Hip**	**BMI**	** **	** **	
SAT/(IMAT+SAT)	-	.40*** **(< .001)**	** **	.43*** **(< .001)**	-.31*** **(< .001)**			
IMAT/(IMAT+SAT)	** **	**.40** *** **(< .001)**	** **	**.31** *** **(< .001)**	.31*** **(< .001)**			
SAT		**.28** *** **(< .001)**	** **	**.28** *** **(< .001)**	.28*** **(< .001)**			
IMAT		**.52** *** **(< .001)**	** **	.14 (< .09)	.46*** **(< .001)**			
Cross-sectional area MM	** **	**.24** ** **(.002)**	** **	**.49** *** **(< .001)**	.49*** **(< .001)**			
Fat fraction		**.24** ** **(.002)**	** **	.07 (.4)	.13 (.1)			

Pearson correlation coefficients (r) and associated p-values are shown in parentheses.

adiponectin (ADPN), body mass index (BMI), cholesterol (Chol), high-density lipoprotein (HDL), intramuscular adipose tissue (IMAT), low density lipoprotein (LDL), muscle mass (MM), subcutaneous adipose tissue (SAT)

*p < .05

**p < .01

***p < .001; n = 156.

Serum adiponectin levels showed a significant positive correlation with the percentage of SAT of the total thigh AT (SAT/(IMAT+SAT)) (cc = 32, p = 001) for the entire study population. (SAT/(IMAT+SAT)) also indicated a significant negative correlation with the cholesterol/HDL ratio (cc = -.36, p = .001) and a significant positive relationship with serum HDL (cc = .33, p < .001). Further, negative correlations were found between serum triglycerides TG (cc = -.27, p = .001) and LDL (cc = -.23, p = .004). Contrary to that of the amount of SAT, which showed relationship with serum adiponectin (cc = .3, p = .001), the amount of IMAT alone did not show a significant correlation with serum adiponectin levels. (cc = -.05, p = .6). In contrast to adiponectin, leptin did not show significant correlations with any of the quantified fat parameters. A weak correlation was found with the cross-sectional area, MM (Table 2).

A separate evaluation of female and male subjects confirmed the relationship between percentage fat distribution of SAT and IMAT (SAT/(IMAT+SAT)) and adiponectin levels in both subgroups (cc = -.30 female, p = .009; cc = -.24 male; p = .036). The amount of SAT or IMAT alone did not correlate with adiponectin in the sex-separated analysis (Table 3).

**Table 3:** Sex-stratified comparison of correlation coefficients between adiponectin and fat tissue segmentation results. The ratio between subcutaneous adipose tissue and intramuscular adipose tissue (SAT/(SAT+IMAT) shows a significant correlation with serum adiponectin levels for both males and females, whereas the correlation of adiponectin levels with either SAT or IMAT alone was not significant for both sexes.

Fat tissue segmentation	Adiponectin	Correlation coefficient	p-value
**SAT/(IMAT+SAT)**	Female	.30	.009***
** **	Male	.24	.036**
**SAT**	Female	.19	.09
** **	Male	.04	.7
**IMAT**	Female	.19	.1
** **	Male	.19	.1

intramuscular adipose tissue (IMAT), subcutaneous adipose tissue (SAT)

*p < .05

**p < .01

***p < .001; n_female_ = 78, n_male_ = 78.

Compared to the male group, the female group showed significantly higher serum adiponectin, and HDL levels (mean±SD) of 12.90±4.71 vs. 10.28±3.07 and 75.94±18.67 vs. 61.94±13.60; whereas, both sexes did not differ in LDL, TG, and total blood cholesterol levels with 103.77±30.29 vs. 103.10±32.36; 108.05±99.04 vs. 122.86±76.87; and 199.53±30.51 vs. 189.51±34.02 mg/dL, respectively (Table 1).

In the multiple linear regression analysis with adiponectin as a constant, we calculated different models including BMI, waist/height, and waist/hip as independent variables and found that the ratio SAT/(SAT+IMAT) demonstrated the highest indicative value for serum adiponectin (p<0.01) (Table 4). BMI and waist/height-ratio showed no significant influence, whereas waist/hip-ratio showed a significant influence (p = .011).

**Table 4:** Effect of anthropometric parameters on the correlation between adiponectin and SAT/(IMAT+SAT).

Predictor	adj. R^2^	F-statistic (p-value)	Coefficient	Estimate	SD	t-statistic	p-value
**Model 1**	.09	**9.14(.000** *** **)**				** **	** **
Constant: **Adiponectin**			***β***_**0**_	0.000	0.076	0.00	1.000
SAT/(IMAT+SAT)			***β***_**1**_	**0.289**	**0.080**	**3.59**	**.000** ***
BMI			***β***_**2**_	0.028	0.080	1,10	.275
**Model 2**	.09	**8.58 (.000** *** *)		** **	** **	** **	** **
Constant: **Adiponectin**			***β***_**0**_	0.000	0.076	0.00	1.000
SAT/(IMAT+SAT)			***β***_**1**_	**0.301**	**0.084**	**3.59**	**.000** ***
Waist/Height			***β***_**2**_	0.037	0.084	0.44	.662
**Model 3**	.13	**12.16 (.000** *** **)**		** **	** **	** **	** **
Constant: **Adiponectin**			***β***_**0**_	0.000	0.075	0.00	1.000
SAT/(IMAT+SAT)			***β***_**1**_	**0.225**	**0.083**	**2.71**	**.007** **
Waist/Hip			***β***_**2**_	**0.214**	**0.083**	**2.58**	**.011** *

*Multiple linear regression coefficients (t- values) and associated p-values are shown*. *Constant*: *adiponectin in all the three models*

***Model*: *y* = *β***_**0**_ **+*β***_**1**_ ***x***_**1**_ **+*β***_**2**_ ***x***_**2**_

subcutaneous adipose tissue (SAT), intramuscular adipose tissue (IMAT)

*p < .05

**p < .01

***p < .001; n = 156.

An overview of the information collected in the questionnaire on pre-existing cardio-metabolic risk factors in our cohort is shown in Table 5.

**Table 5:** Characteristics of the included subjects with cardiovascular risk factors.

**Male**	n = 13
**Smoker**	n = 13
**Hypertension**	n = 7
**Obesity**	n = 8
**Coronary artery disease**	n = 2
**Cardial arrythmia**	n = 2

The differences between the groups with and without history of cardio-metabolic risk factors are indicated in Table 6. There was a significant higher factor (SAT/(SAT+IMAT)) or percentage of IMAT compared to SAT in the group with present cardio-metabolic risk factors (p = .04). Also significant higher serum LDL (p = .005) and lower HDL (p < .001) levels could be found in the group with known risk factors like hypertension or smoking (Table 6).

**Table 6:** Group comparison using between subjects with pre-existing cardiovascular risk factors in history. **A statistically significant difference concerning the** ratio between subcutaneous adipose tissue and intramuscular adipose tissue (SAT/(SAT+IMAT) could be indicated between the two groups using Kruskal-Wallis-Test. Values are shown in (mean ± SD).

	No cardiovascular risk factors (n = 128)	Cardiovascular risk factors (n = 28)	p-value
**SAT/(IMAT+SAT)**	0.85 ± 0.05	0.89 ± 0.05	.04*
**Adiponectin(μg/ml)**	10.88 ± 5.38	12.04 ± 3.84	.15
**Total blood cholesterol (mg/dL)**	191.51 ± 33.33	203.09 ± 33.94	.05*
**HDL (mg/dL)**	71.04 ± 17.19	55.53 ± 14.42	.001***
**Quot(Chol/HDL)**	2.88 ± 0.92	3.85 ± 1.42	.001***
**LDL (mg/dL)**	100.33 ± 30.77	118.03 ± 32.16	.005**

intramuscular adipose tissue (IMAT), subcutaneous adipose tissue (SAT)

*p < .05

**p < .01

***p < .001; n_female_ = 78, n_male_ = 78.

A visual abstract providing an overview of the study and its most important results had been added (S1 Fig).

## Discussion

A connection between SAT amount and serum adiponectin levels as a protective factor of cardiovascular health has already been described [27], as is the role of IMAT as an indicator of insulin resistance and its independent relationship with factors of metabolic syndrome [30,31,35,45]. Our study indicates a significant relationship between serum adiponectin levels and the percentage of SAT of the whole thigh AT (SAT/(SAT+IMAT)), which has to our best knowledge not described in that way in the literature before. In particular, the correlation in both groups of sex should be emphasized when considering the differences in body composition between men and women in terms of fat distribution. Hence, the percentage AT distribution in the thigh was related to serum adiponectin levels and could therefore be an additional indicator for a person’s individual cardiometabolic risk factors. Dube et al. [46] showed a negative association between fatty muscle infiltration and serum adiponectin, but only adjusted for VAT and muscle mass in postmenopausal women. These observations may reinforce the presumption that fatty muscle infiltration is related to insulin resistance and lower serum adiponectin levels but does not explain the prevailing influence of IMAT and SAT on adiponectin levels.

Additionally, the percentage AT distribution in the mid-thigh showed the highest correlation with serum adiponectin level in multilinear regression analysis, in a model including BMI, waist/hip, and waist/height ratios were independent factors. This is a surprising result, as adiponectin secretion takes place to a certain extent in visceral fat and central SAT depots, as demonstrated in in vitro studies [47,48]. These studies showed a dependence of adiponectin secretion, especially in VAT tissue, on BMI [47,48].

The amount of thigh SAT also correlated with serum adiponectin levels in our study (cc = .30, p = .001), as described in previous publications [12,25,27]. However, this relationship was not comprehensible in our evaluation of the sex-stratified analysis. In addition, serum lipids, such as HDL, LDL, and total blood cholesterol, were not significantly related to the amount of SAT in the mid-thigh. As previous studies have shown, women generally have higher serum adiponectin levels than men and relatively higher thigh SAT [49,50]. The reason for this might be that women not only have higher percentages of SAT but also a larger share of IMAT. If one considers the amount of IMAT to be an independent cardiovascular risk factor, as shown in other studies [30,31,34,35], this might relativize the positive effects of a higher proportion of SAT in relation to serum adiponectin levels.

The exact underlying mechanisms, why the distribution of SAT and IMAT relates to serum adiponectin level in both groups of sex, cannot be completely clarified at present. The amount of SAT in the thigh area is positively related to serum adiponectin level because adiponectin is not only produced in the adipocytes of the central, but also in the peripheral subcutaneous tissue [27]. Too much IMAT in relation to SAT has a negative effect independent of BMI, as the amount of IMAT alone has shown to be related to cardiovascular risk-factors and insulin resistance itself [31,34,45].

We assume that too much fat storage in the muscle reduces the insulin-sensitizing effect of adiponectin on skeletal muscle tissue perhaps due to impaired signal transduction at the AdipoR1 receptor [51]. Adiponectin receptor expression is variable between different tissues and also dependent of sex, age and energy intake [38].

In our study, the factor (SAT/(SAT+IMAT)) also correlated negatively with the ratio of cholesterol to HDL (cc = -.36, p = .001) and positively correlated with serum HDL (cc = .33, p = .001). These findings and the positive correlation between serum HDL and adiponectin levels support the observations reported in a study by Wang et al. [52]. An explanation for these connections might be the proposed anti-atherogenic mechanisms of adiponectin in cholesterol efflux and HDL biogenesis via ABCG1 [53,54]. In line with this, inverse relationships were shown between SAT/(SAT+IMAT) and LDL (cc = -.23, p = .004) as well as with serum TG (cc = -.27, p = .001). The positive correlation of our proposed ratio with HDL and the negative correlation with LDL also indicates again the connection of the adipose tissue distribution in the mid-thigh to some cardio-metabolic risk factors.

Interestingly, in contrast to adiponectin, serum leptin levels did not correlate with the (SAT/(SAT+IMAT)) ratio. Because adiponectin is more closely associated with atherosclerosis as a protective adipocytokine [55] than leptin, our data suggest that IMAT expansion reflects a cardiovascular aggressive fat distribution type. The noninvasive measurement of unusually large amounts of IMAT using MRI might identify unhealthy, cardiovascular risk increasing, overweight profiles irrespective of sex and age.

We observed also in our study, that the ratio SAT/(SAT+ IMAT) was significantly less favourable in the group of subjects with pre-existing cardiovascular risk factors such as known hypertension, smoking and obesity, compared to the group without risk factors. An unfavorable distribution pattern means, that there is a higher proportion of IMAT in relation to SAT. This supports the theory, that this factor, quantifying fat distribution, is not only associated with serum adiponectin and HDL, but could be an indicator for individual cardio-metabolic risk factors.

There are several reasons for measuring AT in the mid-thigh in addition to the abdominal region. Previous studies have indicated a relationship between the amount of thigh SAT and serum adiponectin [12,32,56]. Furthermore, Fisher et al. showed constantly high adiponectin levels in the gluteal AT in diabetic subjects [57].

Additionally, we sought to investigate the influence of IMAT, which is different from the VAT compartment, and is stated as an important “VAT-like” AT depot, with an independent relationship with cardio-metabolic risk factors [31,34]. Thigh muscles are one of the largest muscle groups of the body; therefore, we chose this region to measure the amount of IMAT [34]. Moreover, IMAT in the thigh muscles was found to be a stronger predictor of cardiovascular risk than IMAT in calf muscles [30]. Moreover, a comparable reference region, which is highly reproducible, can easily be found in the mid-thigh, and the required instructions are easy to follow [43]. Moving organs produce motion artifacts and gradients, which affect the magnetic field homogeneity or measurements; therefore, the abdominal region is less suitable for quantitative analysis.

This study had some limitations. 1) The sex hormone status was not recorded, which could influence adiponectin levels, especially in postmenopausal women. The results should be confirmed in a larger study cohort to allow for subgroup analyses that can shed light on this question. However, the age distribution in the female group suggests that this influence is limited to our study. The naturally existing sex-specific differences in body composition can probably be more balanced if both lipid compartments that play different roles in lipid metabolism are considered together. 2) The quantitative amount of VAT was not measured, as it is not directly possible to adjust for this correlation on the amount of VAT. In vitro studies have shown that adiponectin is produced in both abdominal SAT and in the VAT compartment [47,48]. Reneau et al. also showed both a dependence of adiponectin secretion on adipocytes and the visceral fat on BMI. Nevertheless, we showed in a multilinear regression analysis that (SAT/(SAT+IMAT)) has the highest predictive value for serum adiponectin levels. However, the waist/hip ratio, which seems to be a significant indicator of serum adiponectin level, is an indirect marker of central obesity, whereas BMI and waist /height ratio indicated no significant relationship. Perhaps, we may need to examine the cellular level of adiponectin secretion separately from our overall results, since in this study, we did not carry out any in vitro measurements. Moreover, it would be interesting to perform in vitro studies on adiponectin secretion in thigh AT and IMAT of the thigh muscles in the future. It might be possible that a dependence of the secretion on BMI may be observed in thigh IMAT. 3) This concerns the two-point Dixon MRI method. Advanced methods such as the three- and six-point Dixon methods have recently been developed [58]. Multi-point Dixon sequences are recommended, as they account for magnetic field homogeneity and correct T_2_*-decay. However, these advantages mainly affect the quantification of fat content by determining the FF, and they play only a minor role in the segmentation of the fat content. 4) The blood pressure was not measured in our study cohort. Pre-existing diseases and risk factors such as hypertension, hyperlipidaemia, smoking behavior and lifestyle were collected by means of a questionnaire. 5.) Finally, no database mining or transcriptomic analysis was performed, and we plan to focus on these points in future studies. However, previous studies with transcriptome analysis of ATs in sheep showed high expression of the gene encoding adiponectin, ADIPOQ, which undermined its pivotal role in fat distribution and deposition [59,60].

Furthermore, a novel feature of the new imaging factor is the additional information on intramuscular fat content. This information is crucial for the clinical assessment of the success of lifestyle interventions. As recently published, especially in young patients with patatin-like phospholipase 3 (PNPLA-3) rs738409 gene polymorphism, a successful lifestyle intervention is extremely important [61]. For persons concerned, such an additional factor could be of great significance because it is capable of estimating intramuscular fat, an important parameter for sufficient clinical follow-up. Additionally, such imaging part of a follow-up examination could be used to enhance patient compliance for lifestyle interventions [62,63].

This new imaging marker could be helpful in validating such a therapeutic approach without exposing patients to ionizing radiation. Such an additional imaging factor could be relevant for a large number of people with progredient nonalcoholic fatty liver disease (NAFLD). More so, AT insulin resistance in muscle tissues plays a key role in the development of metabolic and histological abnormalities in obese patients with NAFLD [36]. According to recent estimations, 25.4% of the world population suffers from NAFLD, which may soon become the main reason for liver transplantation because of its creeping course [64] and only a successful lifestyle intervention is the most effective tool to fight against the dangerous consequences of NFALD, namely NASH and liver fibrosis.

In conclusion, to our best knowledge this is the first study, which describes the association of adipose tissue distribution in the mid-thigh obtained via magnetic resonance imaging with serum adiponectin levels in a cohort of volunteers including both sexes. This ratio is not only associated with serum adiponectin levels in females and males; it also indicates the highest significant relationship with serum adiponectin level in a multilinear regression analysis including BMI, waist/hip, and waist/height ratios. This factor could possibly serve as putative indicator for individual factors of a person´s cardiovascular risk profile in future. We could substantiate this observation, as the ratio was less favorable in the group of subjects with preexisting cardio-vascular risk factors.

The scan time for the MR measurement lasts only one minute. This fact and the increasing availability of MRI equipment argue also for this non-invasive imaging indicator. Therefore, an evaluation of our finding as imaging biomarker should be conducted in further eventually longitudinal studies with larger patient cohorts and a complete cardiovascular examination including atherosclerosis status, blood pressure and more clinical risk factors.

## Supporting information

S1 Fig Visual abstract. Shows a visual abstract providing an overview of the study and its most important results.
(PNG)

S1 Table Anonymized raw data sheet which includes all the relevant data presented in this manuscript. (XLSX)

## Abbreviations

AT: Adipose Tissue
BMI: Body mass index
CT: Computed Tomography
FA: Fatty acid
FF: Fat fraction
HDL: High-density lipoprotein
IMAT: Intramuscular Adipose Tissue
LDL: Low-density lipoprotein
MetS: Metabolic Syndrome
MRI: Magnetic Resonance Imaging
MM: Muscle Mass
SAT: Subcutaneous Adipose Tissue
TG: Triglcerides
VAT: Visceral Adipose Tissue
