# Supplementary figures and images for: Distribution of subcutaneous and intermuscular fatty tissue of the mid-thigh measured by MRI—A putative indicator of serum adiponectin level and individual factors of cardio-metabolic risk
Source: PLoS One. 2021 Nov 15;16(11):e0259952. doi: 10.1371/journal.pone.0259952 (PMC8592416; doi:10.1371/journal.pone.0259952)

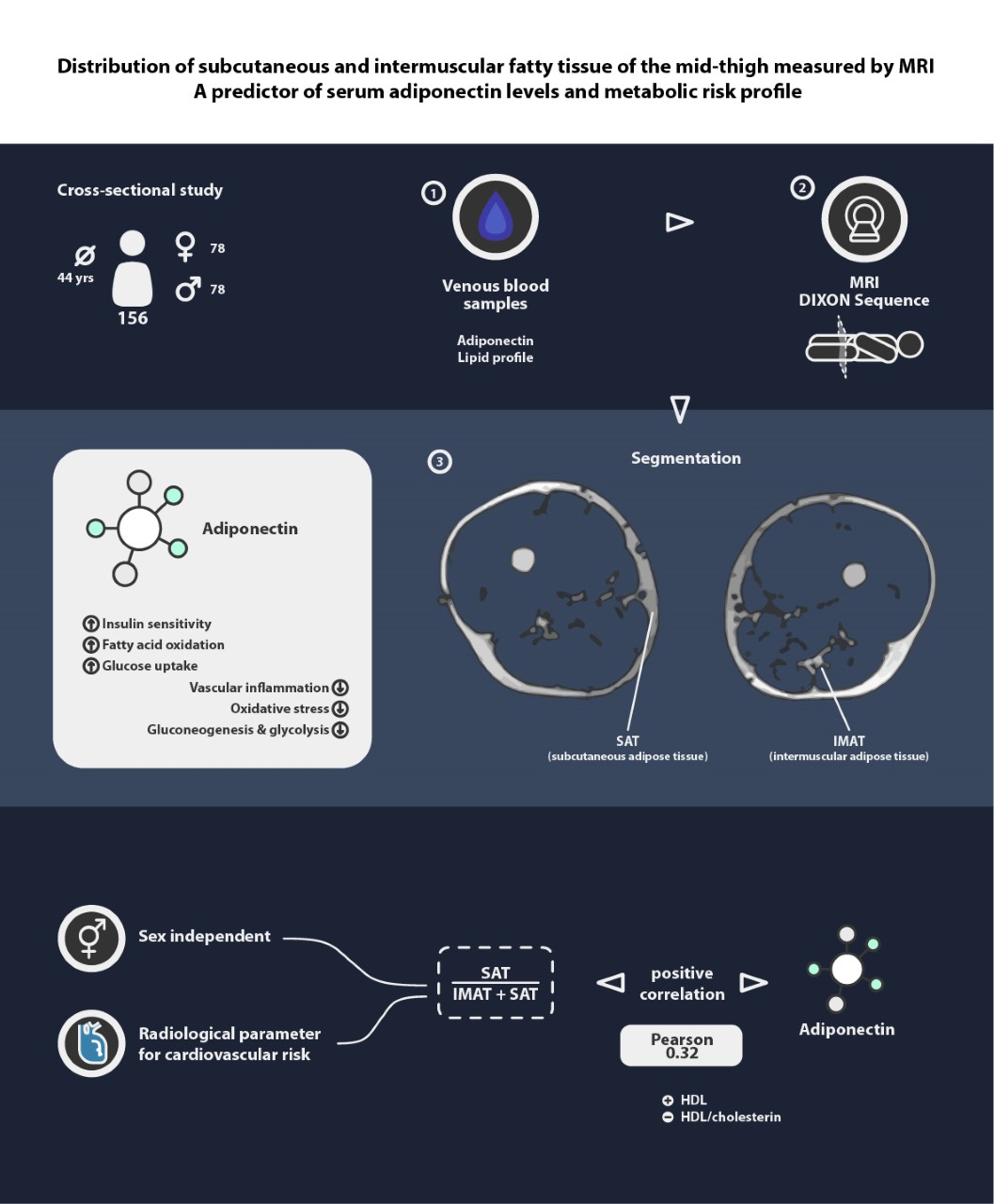

Supplement: S1 Fig — Shows a visual abstract providing an overview of the study and its most important results. (PNG) [file pone.0259952.s001.png]
